# Co-transplantation of pancreatic islets and microvascular fragments effectively restores normoglycemia in diabetic mice

**DOI:** 10.1038/s41536-022-00262-3

**Published:** 2022-11-04

**Authors:** Selina Wrublewsky, Andrea Weinzierl, Isabelle Hornung, Leticia Prates-Roma, Michael D. Menger, Matthias W. Laschke, Emmanuel Ampofo

**Affiliations:** 1 Institute for Clinical & Experimental Surgery, Saarland University, 66421 Homburg, Germany; 2 Biophysics Department, Center for Human and Molecular Biology, Saarland University, 66421 Homburg, Germany

**Keywords:** Type 1 diabetes, Tissue engineering

## Abstract

Insufficient revascularization of pancreatic islets is one of the major obstacles impairing the success of islet transplantation. To overcome this problem, we introduce in the present study a straightforward strategy to accelerate the engraftment of isolated islets. For this purpose, we co-transplanted 250 islets and 20,000 adipose tissue-derived microvascular fragments (MVF) from donor mice under the kidney capsule as well as 500 or 1000 islets with 40,000 MVF into the subcutaneous space of diabetic mice. We found that the co-transplantation of islets and MVF markedly accelerates the restoration of normoglycemia in diabetic recipients compared with the transplantation of islets alone. In fact, the transplantation of 250 islets with 20,000 MVF under the kidney capsule reversed diabetes in 88% of mice and the subcutaneous transplantation of 500 or 1000 islets with 40,000 MVF restored normoglycemia in 100% of mice. Moreover, diabetic mice receiving islets and MVF exhibited plasma insulin levels similar to nondiabetic control animals. Additional immunohistochemical analyses of the grafts revealed a significantly higher number of islet cells and microvessels in the co-transplantation groups. These findings demonstrate that the co-transplantation of islets and MVF is a promising strategy to improve the success rates of islet transplantation, which could be easily implemented into future clinical practice.

## Introduction

Islet transplantation represents a promising therapeutic approach to improve glycometabolic control in type 1 diabetes mellitus (T1DM) patients and in diabetic patients suffering from chronic pancreatitis or following pancreatectomy^1^. Nonetheless, this approach is still not frequently applied in clinical practice, which is mainly due to the rejection as well as the insufficient revascularization and hypoxia-induced failure of grafted islets^2–7^.

The co-transplantation of endothelial cells has been shown to improve the revascularization of tissue grafts^8,9^. However, the reassembly of these cells into new microvessels can take more than 1 week^10^, which is not fast enough to guarantee the survival of the grafts in the initial post-transplant phase. To overcome this problem, an increasing number of studies suggest adipose tissue-derived microvascular fragments (MVF) as suitable vascularization units^11,12^. MVF are a randomized mixture of functional arterioles, capillaries and venules with a length of ~20–200 µm and surrounding single cells, including macrophages and mesenchymal stem cells (MSC)^13–15^. These vessel segments are a rich source of angiogenic growth factors^16,17^. Moreover, they rapidly reassemble into functional microvascular networks and interconnect with the host microvasculature after transplantation into tissue defects^12,18,19^.

Recently, we could demonstrate that the fusion of islet cells with MVF results in compact prevascularized islet organoids^20^. These tissue-engineered organoids are rapidly blood-perfused and, thus, lead to physiological blood glucose levels a few days after transplantation into diabetic mice^20^. Salamone et al.^21^ co-cultivated pancreatic islets and MVF in collagen hydrogels. In their in vitro setting, the MVF formed tubes, branches, and finally, entire capillary networks, which progressively grew into the islets within a week^21^. In line with these results, Aghazadeh et al.^22^ further reported that the embedding of human embryonic stem cell (hESC)-derived pancreatic progenitors into MVF-loaded collagen hydrogels enhances their subcutaneous engraftment and accelerates the reversal of diabetes.

However, the aforementioned tissue engineering approaches require a time-consuming in vitro processing of isolated islets and MVF prior to their transplantation. This may limit their transfer into daily clinical practice. The present study now demonstrates that the simple co-transplantation of a suspension containing a mixture of freshly isolated islets and MVF is similarly effective for the restoration of normoglycemia in diabetic animals and may be much easier implemented into future patient treatment.

## Results

### Cellular composition and viability of isolated islets and MVF

**Fig. 1:**
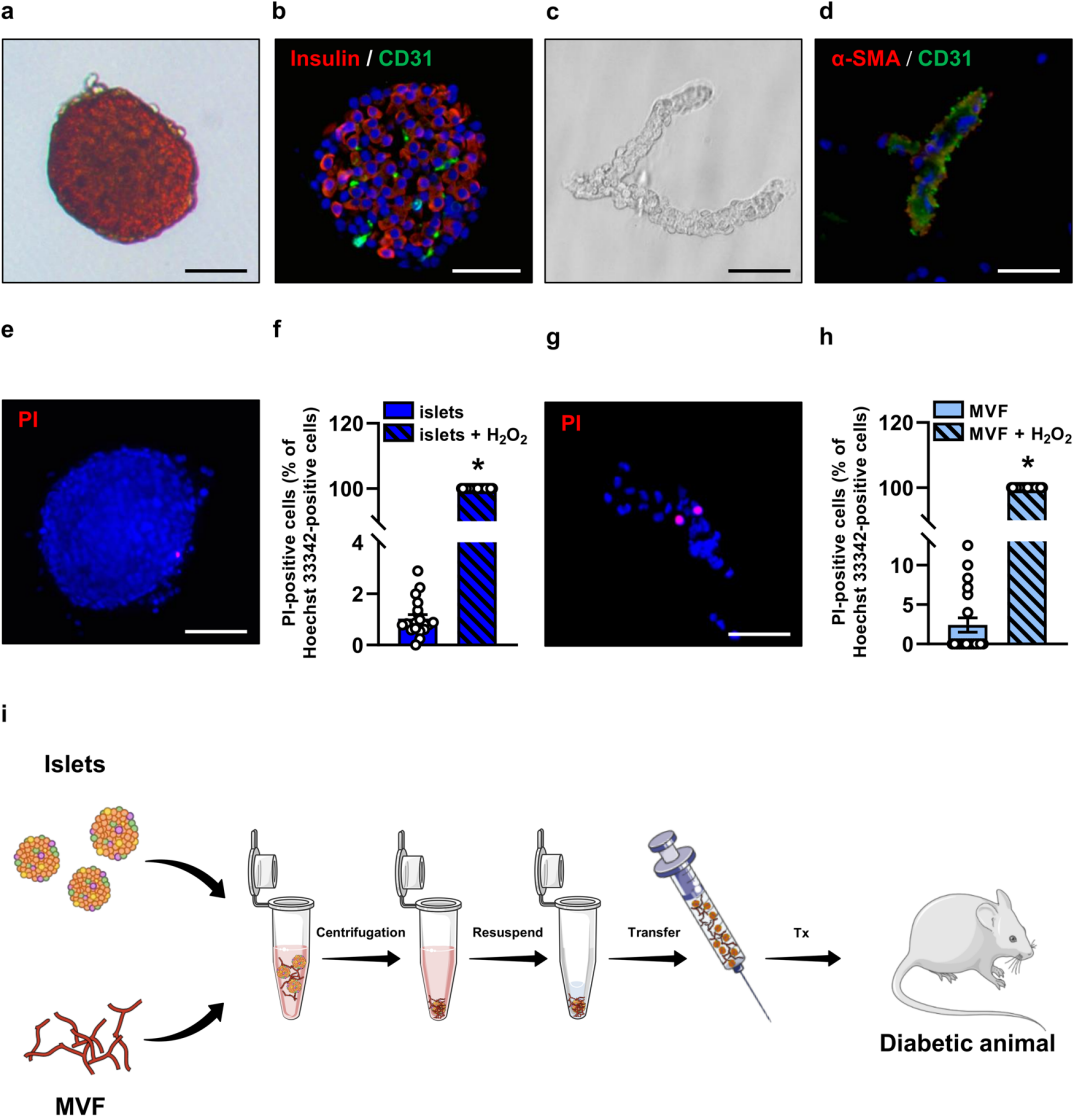
Viability and transplantation of isolated islets and MVF. **a** Bright-field microscopic image of an isolated neutral red-stained islet. Scale bar: 50 μm. **b** Fluorescence microscopic image of insulin (red)- and CD31 (green)-stained islet. Cell nuclei are stained with Hoechst 33342 (blue). Scale bar: 50 μm. **c** Bright-field microscopic image of an isolated MVF. Scale bar: 15 µm. **d** Fluorescence microscopic image of an α-SMA (red)- and CD31 (green)-stained MVF. Cell nuclei are stained with Hoechst 33342 (blue). Scale bar: 15 μm. **e** Fluorescence microscopic image of a PI (red)-stained islet. Cell nuclei are stained with Hoechst 33342 (blue). Scale bar: 100 μm. **f** Quantitative analysis of PI-positive cells (% of all Hoechst 33342-positive cells) within islets directly after their isolation. H_2_O_2_-treated islets served as a positive control. Mean ± SEM (*n* = 20 each group). An unpaired Student’s *t* test was used. ^*^P < 0.05 vs. islets. **g** Fluorescence microscopic image of a PI (red)-stained MVF. Cell nuclei are stained with Hoechst 33342 (blue). Scale bar: 15 μm. **h** Quantitative analysis of PI-positive cells (% of all Hoechst 33342-positive cells) within MVF directly after their isolation. H_2_O_2_-treated MVF served as a positive control. Mean ± SEM (*n* = 20 each group). An unpaired Student’s *t* test was used. ^*^P < 0.05 vs^.^ MVF. **i** Schematic illustration of the co-transplantation of islets and MVF. The isolated islets and MVF were mixed, centrifuged, resuspended in physiological saline solution and transplanted (Tx) under the kidney capsule or into the subcutaneous space of diabetic mice by means of an Hamilton syringe.

Immunohistochemical analyses of freshly isolated islets revealed that they contain a substantial number of CD31-positive endothelial cells (Fig. 1a, b). Isolated MVF were characterized by a CD31-positive endothelium surrounded by α-SMA-positive pericytes (Fig. 1c, d). Only a low number of PI-positive cells were detected in islets and MVF compared with H_2_O_2_-treated controls (Fig. 1e–h), indicating a high viability of the isolates.

### Co-transplantation of isolated islets and MVF under the kidney capsule

To establish a simple approach for the co-transplantation of freshly isolated islets and MVF, we mixed both components in DMEM and concentrated them by centrifugation in a dense pellet (Fig. 1i). This pellet was then resuspended in a small volume of 10 µL physiological saline solution and transferred into a microsyringe (Fig. 1i). Importantly, this approach guaranteed the close contact, and, thus, the direct interaction of islets and MVF after their co-transplantation into diabetic mice (Fig. 1i).

**Fig. 2:**
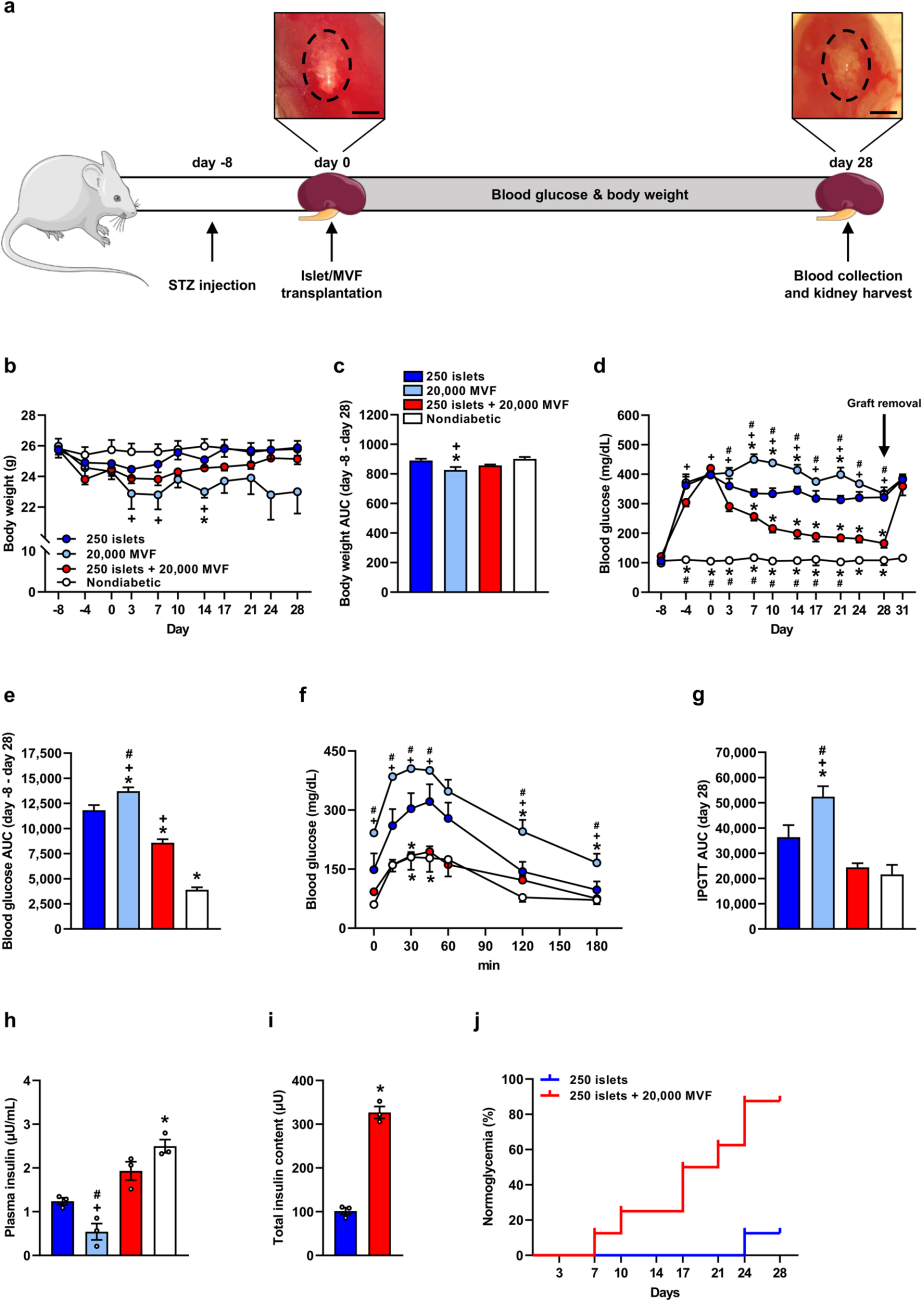
Endocrine function of islets transplanted under the kidney capsule. **a** Schematic illustration of the experimental setting. A diabetic phenotype was induced in mice by a single injection of STZ 8 days prior to islet transplantation. On day 0, 250 islets alone, 250 islets and 20,000 MVF or 20,000 MVF alone were transplanted under the left kidney capsule of the diabetic mice (left image; scale bar: 3 mm). Blood glucose levels and body weights were measured from day -8 to day 31 twice a week. On day 28, the kidneys with the grafts were harvested and blood samples were collected (right image; scale bar: 3 mm). **b** Quantitative analysis of the body weight (g) of mice transplanted with 250 islets alone, 250 islets and 20,000 MVF or 20,000 MVF alone. Nondiabetic mice served as a negative control. Mean ± SEM (*n* = 8 each group). ^*^P < 0.05 vs. 250 islets; ^+^*P* < 0.05 vs^.^ nondiabetic. **c** AUC (day -8–day 28) of the body weights from (**b**). Mean ± SEM (*n* = 8 each group). One-way ANOVA followed by Tukey post hoc test was used. ^*^P < 0.05 vs. 250 islets; ^+^*P* < 0.05 vs. nondiabetic. **d** Blood glucose levels (mg/mL) of diabetic mice transplanted with 250 islets alone, 250 islets and 20,000 MVF or 20,000 MVF alone from day −8 to day 31. On day 28, the grafts were removed by nephrectomy (marked by arrow). Nondiabetic animals served as a negative control. Mean ± SEM (*n* = 8 each group). One-way ANOVA followed by Tukey post hoc test was used. ^*^P < 0.05 vs. 250 islets; ^+^*P* < 0.05 vs. nondiabetic, ^#^*P* < 0.05 vs. 250 islets + 20^,^000 MVF. **e** AUC (day −8 to day 28) of the blood glucose levels from (**d**). Mean ± SEM (*n* = 8 each group). One-way ANOVA followed by Tukey post hoc test was used. ^*^P < 0.05 vs. 250 islets, ^+^*P* < 0.05 vs. nondiabetic, ^#^*P* < 0.05 vs. 250 islets + 20,000 MVF. **f** Quantitative analysis of blood glucose levels (mg/dL) according to the IPGTT of diabetic mice transplanted with 250 islets alone, 250 islets and 20,000 MVF or 20,000 MVF alone. Nondiabetic animals served as negative control. Mean ± SEM (*n* = 8 each group). One-way ANOVA followed by Tukey post hoc test was used. ^*^P < 0.05 vs. 250 islets, ^+^*P* < 0.05 vs. nondiabetic, ^#^*P* < 0.05 vs. 250 islets + 20,000 MVF. **g** AUC (day 28) of the blood glucose levels from (**f**). Mean ± SEM (*n* = 8 each group). One-way ANOVA followed by Tukey post hoc test was used. ^*^P < 0.05 vs. 250 islets, ^+^*P* < 0.05 vs. nondiabetic, ^#^*P* < 0.05 vs. 250 islets + 20,000 MVF. **h** Plasma insulin levels (µU/mL) of diabetic mice transplanted with 250 islets alone, 250 islets and 20,000 MVF or 20,000 MVF alone at 15 min after glucose injection. Nondiabetic mice served as a negative control. Mean ± SEM (*n* = 3 each). One-way ANOVA followed by Tukey post hoc test was used. ^*^P < 0.05 vs. 250 islets, ^+^*P* < 0.05 vs. nondiabetic, ^#^*P* < 0.05 vs. 250 islets + 20,000 MVF. **i** Total insulin content (µU) of the removed grafts from diabetic mice transplanted with 250 islets alone or 250 islets and 20,000 MVF. Mean ± SEM (*n* = 3 each). Unpaired Student’s *t* test was used. ^*^P < 0.05 vs. 250 islets. **j** The proportion of mice that achieved normoglycemia after transplantation of 250 islets alone, 250 islets and 20,000 MVF or 20,000 MVF alone (*n* = 8 each group).

In the first set of experiments, we transplanted a critical number of 250 islets with 20,000 MVF under the kidney capsule of streptozotocin (STZ)-induced diabetic mice. Blood glucose levels and body weights were determined over 28 days (Fig. 2a). Diabetic animals receiving 250 islets or 20,000 MVF alone served as positive controls. At the end of the observation period, the grafts were removed to exclude the spontaneous recovery of pancreatic islets in STZ-treated mice. Nondiabetic animals were used as a negative control. The body weights of nondiabetic animals as well as diabetic animals transplanted with 250 islets or 250 islets and 20,000 MVF did not differ during the 28-day observation period (Fig. 2b, c). In contrast, we measured decreased body weights in animals solely transplanted with 20,000 MVF (Fig. 2b, c). The transplantation of 250 islets or 20,000 MVF alone could not reverse diabetes. Of note, the co-transplantation of 250 islets and 20,000 MVF significantly lowered the blood glucose levels of diabetic mice after only 3 days and normoglycemia was achieved after 14 days (Fig. 2d). Moreover, the area under the curve (AUC) of blood glucose levels of mice receiving 250 islets and 20,000 MVF was also significantly reduced (Fig. 2e). An intraperitoneal glucose tolerance test (IPGTT) on day 28 after transplantation showed that the blood glucose levels of mice co-transplanted with islets and MVF were lower compared with positive controls (Fig. 2f, g). In line with these results, the plasma insulin level and the total insulin content of the grafts were significantly higher (Fig. 2h, i). Of interest, the co-transplantation of 250 islets and 20,000 MVF reversed diabetes in 87.5% of recipients, whereas only 12.5% of mice receiving 250 islets alone were normoglycemic on day 28 (Fig. 2j).

**Fig. 3:**
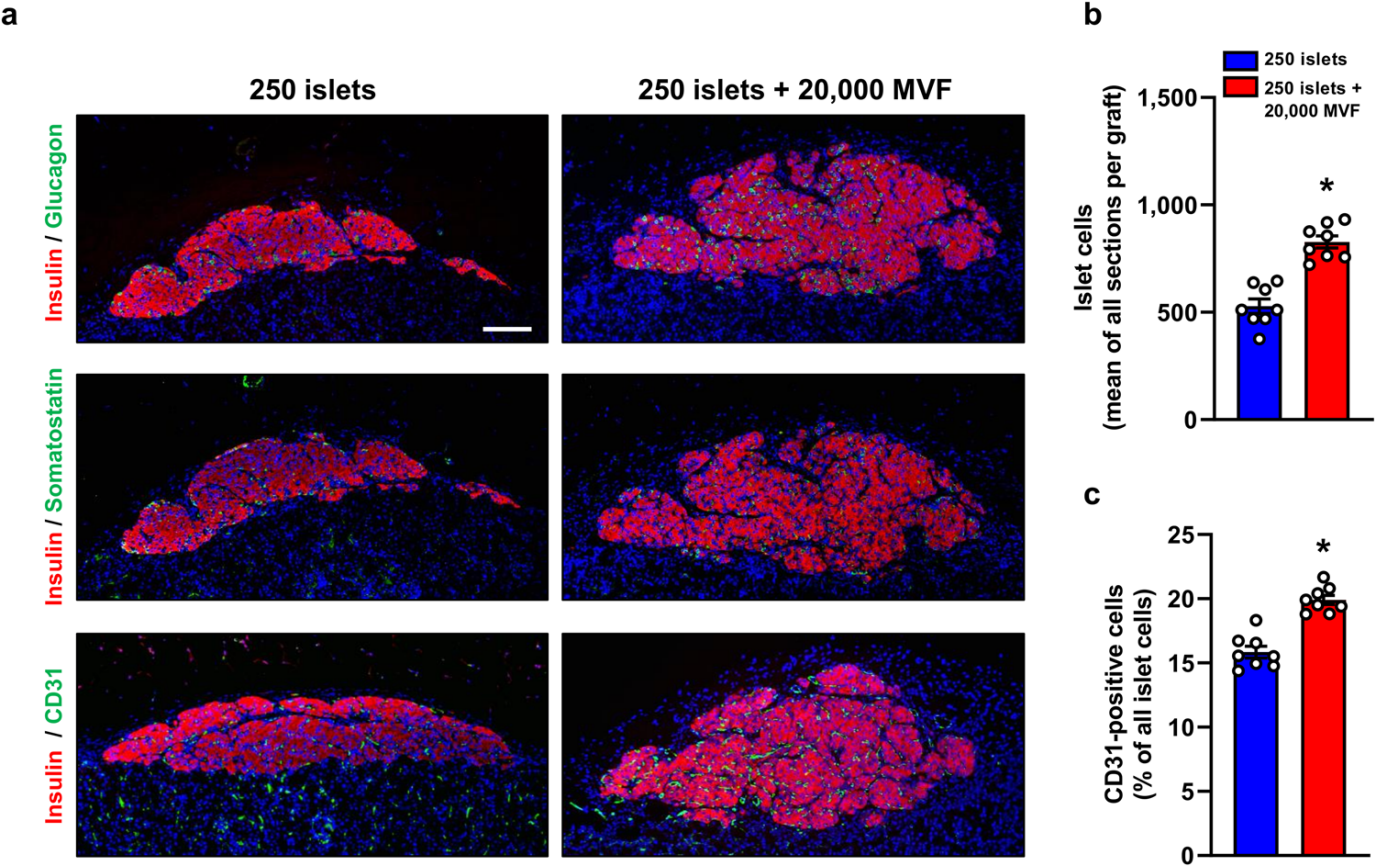
Immunohistochemical analysis of the grafts under the kidney capsule. **a** Immunofluorescent stainings of insulin/glucagon, insulin/somatostatin, and insulin/CD31 within the group of 250 islets alone and the group of 250 islets and 20,000 MVF on day 28 after transplantation. Scale bar: 100 µm. **b** Quantitative analysis of islet cells (mean of all sections per graft) within the group of 250 islets alone and the group of 250 islets and 20,000 MVF. Mean ± SEM (*n* = 8 each group). An unpaired Student’s *t* test was used. ^*^P < 0.05 vs. 250 islets. **c** Quantitative analysis of CD31-positive cells (% of all islet cells) within the group of 250 islets alone and the group of 250 islets and 20,000 MVF on day 28 after islet transplantation. Mean ± SEM (*n* = 8 each group). An unpaired Student’s *t* test was used. ^*^P < 0.05 vs^.^ 250 islets.

At the end of the in vivo experiments, the kidneys of mice receiving 250 islets or a combination of 250 islets and 20,000 MVF were harvested and the grafts’ cellular composition was analyzed by means of immunohistochemistry (Fig. 3a). The fractions of insulin, glucagon and somatostatin-positive cells did not differ between the groups. However, we detected a significantly higher number of islet cells in mice co-transplanted with 250 islets and 20,000 MVF (Fig. 3a, b). As expected, we also determined a higher fraction of CD31-positive endothelial cells in co-transplanted mice (Fig. 3a, c). This indicates that the improved glycometabolic control in diabetic animals is mediated by accelerated graft vascularization.

### Co-transplantation of isolated islets and MVF into the subcutaneous space

**Fig. 4:**
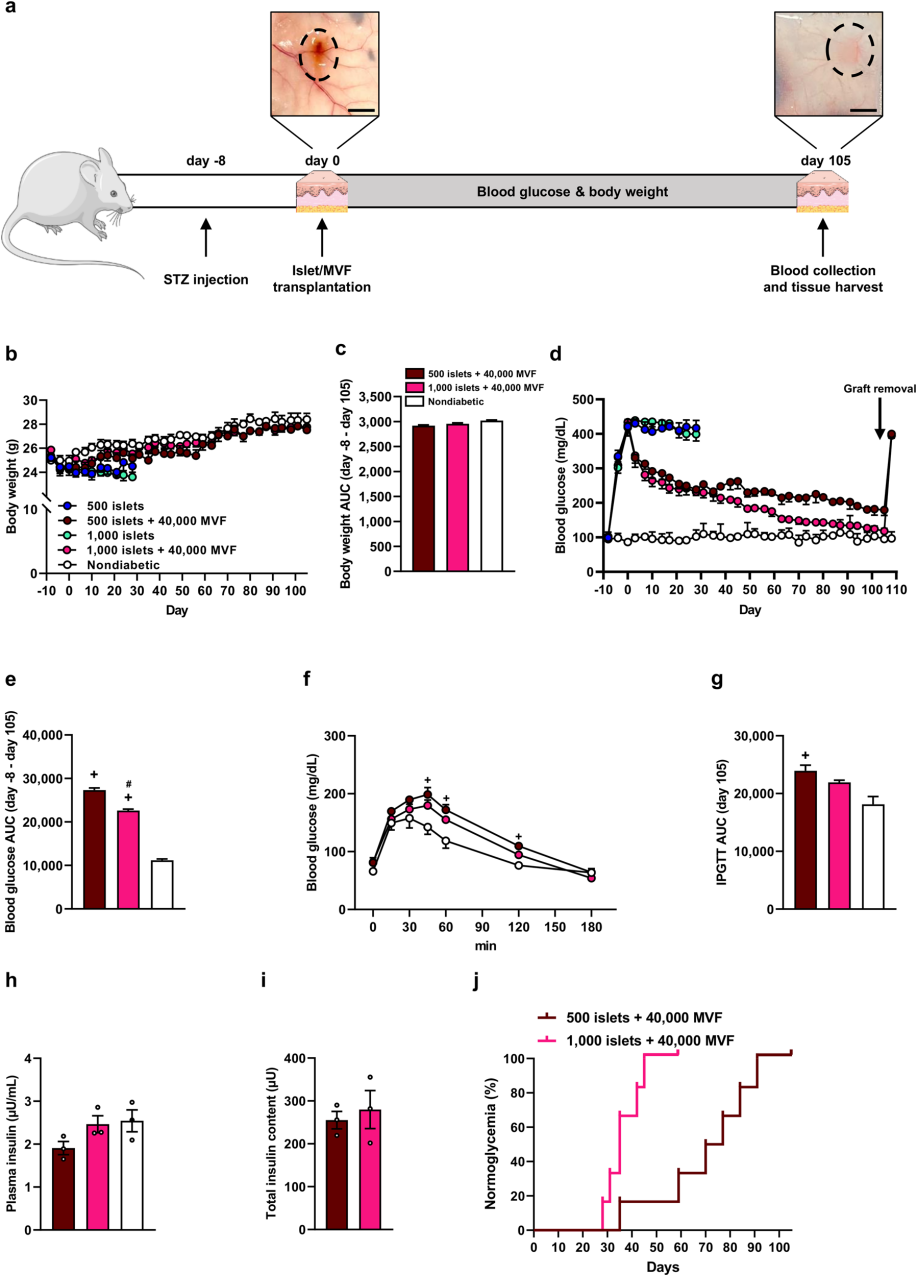
Endocrine function of islets transplanted into the subcutaneous space. **a** Schematic illustration of the experimental setting. A diabetic phenotype was induced in mice by a single injection of STZ 8 days prior to islet transplantation. On day 0, 500 or 1000 islets with or without 40,000 MVF were subcutaneously transplanted into the diabetic mice (left image; scale bar: 2 mm). Blood glucose levels and body weights were measured from day -8 to day 108 twice a week. On day 105, the subcutaneous tissue with the grafts was harvested, and blood samples were collected (right image; scale bar: 2 mm). **b** Quantitative analysis of the body weight (g) of mice transplanted with 500 or 1000 islets with or without 40,000 MVF. Nondiabetic mice served as a negative control. Mean ± SEM (*n* = 6 each group). **c** AUC (day −8 to day 105) of the body weights from (**b**). Mean ± SEM (*n* = 6 each group). **d** Blood glucose levels (mg/mL) of diabetic mice transplanted with 500 or 1000 islets with or without 40,000 MVF. On day 105, the grafts were removed (marked by arrow). Nondiabetic animals served as a negative control. Mean ± SEM (*n* = 6 each group). **e** AUC (day −8 to day 105) of the blood glucose levels from (**d**). Mean ± SEM (*n* = 6 each group). One-way ANOVA followed by the Tukey post hoc test was used. ^+^*P* < 0.05 vs. nondiabetic, ^#^*P* < 0.05 vs^.^ 500 islets + 40,000 MVF. **f** Quantitative analysis of blood glucose levels (mg/dL) according to the IPGTT of diabetic mice transplanted with 500 or 1000 islets with 40,000 MVF. Nondiabetic animals served as a negative control. Mean ± SEM (*n* = 6 each group). One-way ANOVA followed by Tukey post hoc test was used. ^+^*P* < 0.05 vs. nondiabetic. **g** AUC (day 105) of the blood glucose levels from (**f**). Mean ± SEM (*n* = 6 each group). One-way ANOVA followed by Tukey post hoc test was used. ^+^*P* < 0.05 vs. nondiabetic. **h** Plasma insulin levels (µU/mL) of diabetic mice transplanted with 500 or 1000 islets with 40,000 MVF at 15 min after glucose injection. Nondiabetic mice served as a negative control. Mean ± SEM (*n* = 3 each). **i** Total insulin content (µU) of the removed grafts from diabetic mice transplanted with 500 or 1000 islets with 40,000 MVF. Mean ± SEM (*n* = 3 each). **j** The proportion of mice that achieved normoglycemia after transplantation of 500 or 1000 islets with 40,000 MVF (*n* = 6 each group).

In the second set of experiments, we focused on the subcutaneous space as a potential clinically applicable transplantation site for islets^23,24^. For this purpose, 500 or 1000 islets were co-transplanted with 40,000 MVF. Mice transplanted with 500 or 1000 islets alone served as positive controls, and nondiabetic mice served as a negative control. We followed graft function for >100 days with the exception of mice receiving 500 or 1000 islets, because most of these animals had to be sacrificed within the first 28 days after transplantation due to the severity of diabetes (Fig. 4a). We detected no differences in the body weights between the animals of the different groups during the 105-day observation period (Fig. 4b, c). As expected, the transplantation of 500 or 1000 islets alone did not restore normoglycemia (Fig. 4d). Of interest, the co-transplantation of 500 or 1000 islets with 40,000 MVF markedly reduced hyperglycemia (Fig. 4e). More importantly, at the end of the observation period we measured physiological blood glucose levels in mice receiving 1000 islets with 40,000 MVF that were similar to those of nondiabetic mice (Fig. 4d). An IPGTT on day 105 after transplantation showed that the blood glucose levels of mice co-transplanted with 1000 islets and 40,000 MVF were slightly lower compared with animals co-transplanted with 500 islets and 40,000 MVF (Fig. 4f, g). We additionally determined an elevated plasma insulin level and total insulin content of the removed grafts in the group of 1000 islets and 40,000 MVF compared with the group of 500 islets with 40,000 MVF (Fig. 4h, i). As expected, the two groups reversed diabetes in 100% of recipients (Fig. 4j).

**Fig. 5:**
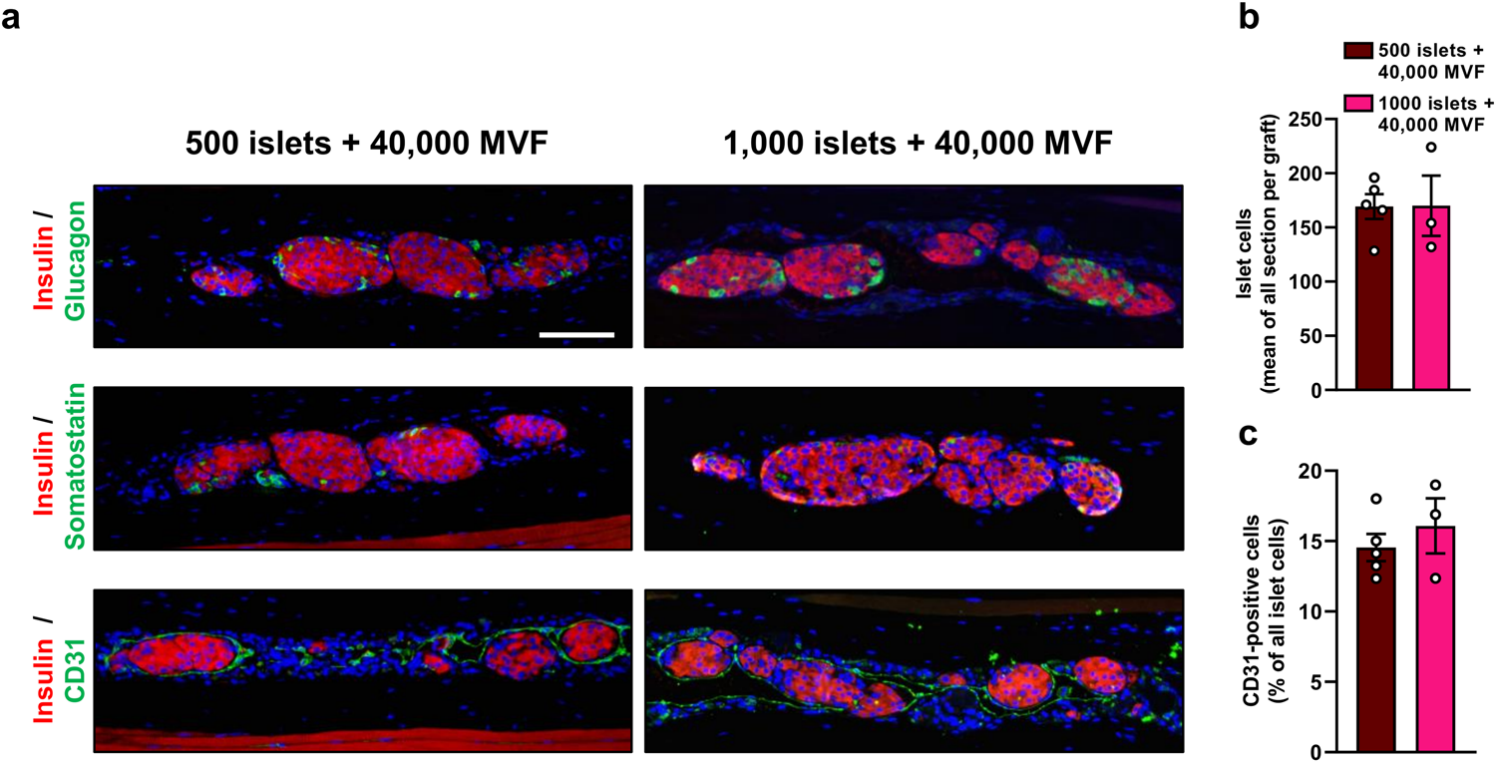
Immunohistochemical analysis of the grafts in the subcutaneous space. **a** Immunofluorescent stainings of insulin/glucagon, insulin/somatostatin, and insulin/CD31 within the group of 500 or 1000 islets with 40,000 MVF on day 105 after subcutaneous transplantation. Scale bar: 100 µm. **b** Quantitative analysis of islet cells (mean of all sections per graft) within the group of 500 (*n* = 5) or 1000 (*n* = 3) islets with 40,000 MVF. Mean ± SEM. **c** Quantitative analysis of CD31-positive cells (% of all islet cells) within the group of 500 (*n* = 5) or 1000 (*n* = 3) islets with 40,000 MVF. Mean ± SEM.

Finally, grafts from mice co-transplanted with 500 or 1000 islets and 40,000 MVF were harvested on day 105 for additional immunohistochemical analyses. The grafts contained β-cells, α-cells, and δ-cells, as shown by positive insulin, glucagon and somatostatin stainings (Fig. 5a). The overall number of islet cells and the fraction of CD31-positive cells did not differ between the groups (Fig. 5b, c). All these results clearly indicate that the co-transplantation of islets with MVF markedly improves the restoration of normoglycemia in diabetic animals by enhancing islet engraftment.

## Discussion

In a previous proof-of-concept study, we have introduced a novel MVF-based strategy to improve the outcome of islet transplantation^20^. For this purpose, we generated prevascularized islet organoids by the fusion of pancreatic islet cells with MVF. These organoids exhibited a highly angiogenic activity, which was mediated by a paracrine signaling between β-cells and endothelial cells. Moreover, we found that the transplantation of a critical number of 250 islets under the kidney capsule does not restore normoglycemia in diabetic animals, whereas the transplantation of an identical number of prevascularized islet organoids leads to physiological blood glucose levels after transplantation^20^. However, although this approach was successful, the generation of prevascularized organoids may be difficult to be implemented into clinical practice due to complex and time-consuming culture procedures. To overcome this problem, we herein introduce a much simpler MVF-based strategy. In fact, the present study clearly shows that the transplantation of a mixture containing freshly isolated MVF and islets markedly accelerates graft revascularization. This is not only crucial for the metabolic response of the islet grafts to glucose, but also guarantees their survival at poorly vascularized transplantation sites, such as the subcutaneous space^23–25^.

MVF are potent vascularization units, which rapidly interconnect with each other and the surrounding host microvasculature after transplantation into new blood-perfused networks^12,13,15,26,27^. Accordingly, we detected much more microvessels within islet grafts co-transplanted with MVF under the kidney capsule compared with controls. In line with this observation, these grafts also finally exhibited a higher number of islet cells. This clearly indicates the relevance of an adequate revascularization of transplanted islets for their long-term survival and function^20,25,28^.

Several studies reported that intravenous transplantation of MSC after STZ injection protects endogenous β-cells within the pancreas from cell death^29,30^. However, it is still ambiguous whether this protective effect is mediated by the direct cell-cell contact between MSC and β-cells or by soluble factors secreted from these stem cells^31^. Of note, MVF also contain a substantial number of MSC^32^. Therefore, we transplanted 20,000 MVF alone under the kidney capsule as an additional control group. By this, we could exclude the previously described positive effect on endogenous β-cells in STZ-induced diabetic mice. Indeed, we found that the grafted MVF did not affect hyperglycemia during the entire in vivo observation period.

Furthermore, it should be noted that MSC can rescue transplanted islets from hypoxia-induced cell death by promoting the establishment of a new blood supply and reducing inflammation^33^. Detailed analyses showed that this is mediated by enhanced secretion of angiogenic factors, including hepatocyte growth factor (HGF) and angiopoietin-1 (Ang-1), from MSC^33^. Therefore, it is conceivable that in the present study the improved islet engraftment is not only driven by the reassembly of co-transplanted MVF into new microvascular networks but also by soluble angiogenic factors from MVF-derived MSC.

The kidney capsule is frequently used as an experimental islet transplantation site in rodents^34^, whereas it is not used in patients due to common diabetes-related renal complications^35^. In contrast, the subcutaneous transplantation site has appealing potential for clinical use, because it exhibits a large surface area and is easily accessible for graft placement, monitoring and possible retrieval. However, many experimental studies have already demonstrated that only a high number of islets (≥1000) subcutaneously transplanted in diabetic mice reverses hyperglycemia^23,36–39^. This can be explained by the poor vascularization and low tissue oxygen tension of the subcutaneous space compared with other transplantation sites, such as the kidney capsule^38,40^.

Therefore, various research efforts have been made to improve the engraftment of islets by increasing the microvascular density of the subcutaneous tissue^37^ or by embedding islets into carrier gels prior to their transplantation^41^. For the latter approach, collagen hydrogels have been shown to be suitable, because they promote the expression of extracellular matrix proteins and are characterized by a low immunogenicity^42^. Recently, Aghazadeh et al.^22^ reported that the embedding of 8000 MVF and 3 × 10^6^ human embryonic stem cell (hESC)-derived pancreatic progenitors within collagen hydrogels and their subsequent transplantation into a subcutaneous pocket maintains long-term normoglycemia in immunocompetent mice. In our study, we demonstrated that the co-transplantation of 500 murine islets and 40,000 MVF restores normoglycemia (≤200 mg/dL). Of note, the same number of MVF co-transplanted with 1000 islets even led to blood glucose levels similar to nondiabetic animals. In contrast, most of the animals receiving islets alone died ~30 days after transplantation due to the severity of diabetes. The lower MVF to islet ratio to sufficiently reverse hyperglycemia in the study of Aghazadeh et al. ^22^ may be explained by the positive effects of the used collagen hydrogel carrier. In fact, Llacua et al.^43^ showed that exogenous collagen promotes the viability of isolated islets in alginate-based microcapsules. Moreover, MVF cultured in collagen hydrogel expressed increased mRNA levels of vascular endothelial growth factor (VEGF) and platelet-derived growth factor (PDGF)^21^. However, collagen hydrogels also bear several disadvantages. Dependent on their fabrication, they are highly variable in their mechanical, structural and transport properties, which aggravates their standardization and comparability in different studies^44^. Moreover, they act as a barrier increasing the initial spatial separation of islets and MVF. This may prolong the critical time period during which the islet grafts are solely supplied with oxygen and nutrients via diffusion, resulting in a high rate of graft failure. For these reasons, a standardized and effective islet transplantation strategy based on collagen hydrogels may be difficult to implement into clinical practice.

Taken together, we herein established a straightforward approach to accelerate islet engraftment by the co-transplantation with MVF. This approach is highly effective, as it only requires a low number of subcutaneously transplanted islets for the restoration of normoglycemia in diabetic mice. Moreover, it may be easily transferred into future clinical practice, because it circumvents complex and time-consuming tissue engineering strategies.

## Methods

### Reagents

Collagenase NB 4G was purchased from SERVA Elektrophoresis GmbH (Heidelberg, Germany). Collagenase NB 8 Broad Range was purchased from Nordmark Biochemicals (Uetersen, Germany). Dulbecco’s Modified Eagle’s Medium (DMEM) was purchased from Thermo Fisher Scientific (Karlsruhe, Germany). Hoechst 33342, neutral red solution, penicillin, and STZ were purchased from Sigma-Aldrich (Taufkirchen, Germany). HepatoQuick^®^ was purchased from Roche (Basel, Switzerland). Propidium iodide (PI) was purchased from BD Biosciences (San Jose, CA, USA). Ketamine (Ursotamin^®^) was purchased from Serumwerke Bernburg (Bernburg, Germany). Xylazine (Rompun^®^) was purchased from Bayer (Leverkusen, Germany).

### Antibodies

The anti-CD31 antibody (DIA310; 1:300) was purchased from Dianova (Hamburg, Germany). The anti-α-smooth muscle actin (SMA) (Ab5694; 1:500), anti-insulin (Ab7842; 1:300), anti-somatostatin (Ab30788; 1:300) and anti-glucagon (Ab92587; 1:300) antibodies were purchased from Abcam (Cambridge, UK). The goat-anti-rabbit-cyanine (Cy)3 (1:1000); goat-anti-mouse-Cy3 (1:1000); goat-anti-rat-Cy3 (1:1000); goat-anti-rat-AlexaFluor555 (1:1000) and goat-anti-rabbit-streptavidin-peroxidase (1:500) antibodies were purchased from GE Healthcare (Freiburg, Germany).

### Animals

Male and female C57BL/6N mice with an age of 10–16 weeks and a body weight of 25–30 g served as donors for islet isolation. Male C57BL/6N mice with an age of 7–12 months and a body weight of 30–35 g served as donors for MVF isolation. Diabetes was induced in 6–8-week old male C57BL/6N mice with a body weight of 24–28 g. The animals were maintained on a standard 12/12 h day/night cycle. Water and standard pellet chow (Altromin, Lage, Germany) were provided ad libitum. All experiments were approved by the local governmental animal protection committee (Landesamt für Verbraucherschutz, Abteilung C Lebensmittel- und Veterinärwesen, Saarbrücken, Germany) and were conducted in accordance with the European legislation on protection of animals (Guide line 2016/63/EU) and the National Institutes of Health Guidelines for the Care and Use of Laboratory Animals (http://oacu.od.nih.gov/regs/index.htm, eighth edition, 2011).

### Isolation of pancreatic islets

Mice were anesthetized by intraperitoneal (i.p.) injection of ketamine (100 mg/kg body weight) and xylazine (12 mg/kg body weight). Following cervical dislocation and midline laparotomy, the pancreatic duct was injected with 1 mg/mL collagenase NB 8 containing 25 µL/mL neutral red solution. The pancreas is excised and further digested by collagenase (collagenase NB 4G; 1 mg/mL). After washing with phosphate-buffered saline (PBS) containing 10% fetal calf serum (FCS) to inactivate the collagenase, the islets were purified by hand-picking. Islets were then transferred in DMEM (supplemented with 10% (v/v) FCS, 100 U/mL penicillin and 0.1 mg/mL streptomycin). For the in vitro and in vivo experiments, we used islets in a narrow size range of ~120–200 µm.

### Isolation of MVF

For the isolation of MVF, mice were anesthetized by an i.p. injection of ketamine (100 mg/kg body weight) and xylazine (12 mg/kg body weight). The epididymal fat pads were harvested, washed and mechanically dissected before the tissue was enzymatically digested by collagenase NB 4G for 10 min. After their isolation, MVF were transferred in DMEM (10% (v/v) FCS, 100 U/mL penicillin and 0.1 mg/mL streptomycin).

### PI staining

To analyze the number of dead cells, isolated islets and MVF were incubated for 10 min with 2 μg/mL PI and 2 μg/mL Hoechst 33342 at RT. Islets and MVF incubated for 24 h with 0.2% H_2_O_2_ served as positive controls. The stained cells were transferred to a slide, sealed with a coverslip, and visualized by fluorescence microscopy (BX60 microscope; Olympus). The number of PI-positive cells was given in % of all Hoechst 33342-positive cells.

### Diabetes induction and islet transplantation

Diabetic phenotypes were induced by a single i.p. injection of 180 mg/kg STZ 8 days prior to islet transplantation. Body weights and non-fasting blood glucose levels of STZ-injected mice were measured twice a week during the entire observation period of 28 or 105 days. Blood samples were taken from the tail vein and analyzed by a portable blood glucose monitoring system (GL50; Breuer, Ulm, Germany). Mice with a non-fasting blood glucose level ≥350 mg/dL served as diabetic recipients for islet transplantation.

In a first set of experiments, a mixture of 250 islets and 20,000 MVF were injected under the left kidney capsule of diabetic mice. For this, a small nick was made in the kidney capsule with the bevel of a 10 µl Hamilton syringe over the inferior renal pole. Islets and MVF are then deposited under the capsule through the nick towards the superior pole of the kidney. The kidney is returned to the retroperitoneal space and the incisions are closed. Diabetic animals receiving 250 islets or 20,000 MVF alone served as positive controls. Whereas nondiabetic animals, which did not receive islets and MVF were used as a negative control.

In another set of experiments, a mixture of 500 or 1000 islets with 40,000 MVF were injected into the subcutaneous space of diabetic mice. For this purpose, a 24G needle (Braun, Melsungen, Germany) was used to penetrate the skin on the medial dorsum of the animal. After removing the needle, the islets and MVF were transplanted by means of a 10 µL Hamilton syringe. Diabetic animals receiving 500 or 1000 islets alone served as positive controls. Nondiabetic animals, which did not receive islets, were used as a negative control. Normoglycemia was defined by blood glucose levels ≤200 mg/dL.

To confirm graft-dependent normoglycemia, the islet transplants were explanted either by nephrectomy or subcutaneous graft excision. For this, the graft-bearing kidney was exposed and the renal vessels and the ureter were ligated at the pedicle. Thereafter, the organ was explanted. The subcutaneous islet grafts were excised with a margin of the surrounding skin. Thereafter, blood glucose levels were monitored for the subsequent 3 days to observe a return to hyperglycemia.

### IPGTT and insulin enzyme-linked immunosorbent assay (ELISA)

An IPGTT was performed on day 28 or 105 after islet transplantation. For this purpose, the mice were i.p. injected with a 10% glucose solution after 16 h of fasting. Subsequently, blood glucose levels were determined at 0, 15, 30, 45, 60, 120 and 180 min using blood from the tail vein and analyzed by a portable blood glucose monitoring system (GL50; Breuer).

Additional mice were anesthetized 15 min after glucose injection and blood samples were collected from the vena cava. The blood plasma was separated by centrifugation and the plasma insulin levels were analyzed by means of an insulin ELISA kit (Invitrogen, USA) according to the manufacturer’s protocol.

To determine the total insulin content of the grafts, the islet transplants underneath the kidney capsule and inside the subcutaneous tissue were dissected, lysed in 1 mL RIPA lysis buffer and the intracellular insulin content was determined by an insulin ELISA kit according to the manufacturer’s protocol.

### Immunohistochemistry

Isolated islets and MVF (both embedded in HepatoQuick^®^) as well as kidneys and subcutaneous tissue containing the grafts were fixated for 24 h in 4% paraformaldehyde (PFA) at 4 °C. After dehydration, the PFA-fixed samples were embedded in paraffin and 3-μm-thick sections were cut. The sections were stained with antibodies against insulin (1:300), glucagon (1:300), somatostatin (1:300), CD31 (1:300) and α-SMA (1:300) and visualized by their corresponding secondary antibodies. Cell nuclei were stained with Hoechst 33342. The sections were analyzed by means of fluorescence microscopy (BX60 microscope; Olympus). The transplanted islets within the kidney capsules and subcutaneous tissue were serially cut, and every 10th slice was immunohistochemically stained. The quantification of positively stained cells was done per graft by FIJI software (NIH) at a magnification of 100x and is given in mean of all sections per graft or in % of all islet cells per section.

### Statistical analysis

All in vitro experiments were reproduced at least three times. For the in vivo experiments, we used at least five animals per group and no mice were excluded from the statistical analysis. After testing the data for normal distribution and equal variance, differences between the two groups were assessed by the unpaired Student’s *t* test. To test differences between multiple groups, one-way ANOVA was applied. This was followed by the Tukey post hoc test. Statistical analyses were performed by means of Prism software 8 (GraphPad, San Diego, CA, USA). All values are expressed as mean ± SEM. Statistical significance was accepted for *P* < 0.05.

### Reporting summary

Further information on research design is available in the Nature Research Reporting Summary linked to this article.

## Supplementary information

REPORTING SUMMARY

## Data Availability

All data needed to evaluate or reproduce the conclusions in the paper are present in the paper. The data that support the findings of this study are available from the corresponding author.
